# Bone Metastases of Glioblastoma: A Case Report and Review of the Literature

**DOI:** 10.3389/fonc.2021.705455

**Published:** 2021-09-27

**Authors:** Wei Zhang, Yuan-yuan Cai, Xiao-li Wang, Xiao-xiao Wang, Yang Li, Gui-yan Han, Yu-jing Chu, Yun-xiang Zhang, Fu-rong Hao

**Affiliations:** ^1^ Clinical School, Weifang Medical University, Weifang, China; ^2^ Department of Radiation Oncology, Weifang People’s Hospital, Weifang, China; ^3^ Department of Pathology, Weifang People’s Hospital, Weifang, China; ^4^ Department of Imaging, Weifang People’s Hospital, Weifang, China; ^5^ Weifang Key Laboratory of Radiophysics and Oncological Radiobiology, Weifang, China

**Keywords:** glioblastoma, extracranial metastasis, soft tissue metastasis, nerve root, intercostal nerve, case report

## Abstract

**Background:**

Glioblastoma (GBM) is the most common primary intracranial tumor and originates from the small pool of adult neural stem and progenitor cells (NSPCs). According to the World Health Organization (WHO) classification of brain tumors, gliomas are classified into grades I–IV, and GBM is defined as the highest grade (IV). GBM can be disseminated by cerebrospinal fluid (CSF), but extracranial metastasis is rare. Additionally, the pathway and mechanism involved remain unclear.

**Case Presentation:**

We report a rare case of left temporal lobe GBM with multiple bone metastases and soft tissue metastasis. This 49-year-old right-handed man who was diagnosed with GBM underwent surgery on May 9, 2017, followed by radiochemotherapy in June 2017. On August 13, 2019, local relapse was found. Then, the patient received a second surgery but not radiochemotherapy. In November 2019, the patient was reported to be suffering from low back pain for nearly 1 month. On December 6, 2019, magnetic resonance imaging (MRI) of the thoracolumbar vertebrae and abdominal computed tomography (CT) confirmed metastases on the ninth posterior rib on the right, the third anterior rib on the left, and the T7 and T10 vertebrae and their appendages. CT-guided rib space-occupying puncture biopsy was performed, and GBM was identified by pathology.

**Conclusion:**

We should pay attention to extracranial metastasis of GBM. Timely detection and early treatment improve overall quality of patients’ life. The extracranial metastasis in this patient may have occurred through the spinal nerve root or intercostal nerve. Further clinical observations are required to clarify the pathway and mechanism involved.

## Background

Malignant brain tumors are among the most feared types of cancer, not only because of their poor prognosis but also because of their direct repercussions on quality of life and cognitive function. Glioblastoma (GBM) is the most notorious intracranial tumor in adults (1); after standard treatment (surgery, chemotherapy, and radiation therapy), there is still a high rate of locoregional relapse, and the median survival time of GBM patients is only 15 months (2, 3). Peripheral metastases of GBM are very rare. Usually, they occur in regional lymph nodes (51%) and the lungs and pleura (60%) and occasionally in the bone (31%) and liver (22%) (4); those in soft tissues are extremely unusual (5). In addition, the most common site of bone metastasis is the vertebrae (6). Metastatic diffusion of cerebrospinal fluid (CSF) is observed in some cases. Peripheral metastases of GBM are very rare despite the ability of GBM cells to pass through the blood–brain barrier (BBB) and be disseminated throughout the peripheral blood (7). The mechanism of their diffusion is not well known. We report this rare case of multiple metastases of GBM including bone (vertebrae and rib cage) and soft tissues and discuss a possible new mechanism for metastasis/extracranial metastasis of GBM, which may occur through the nerve root or intercostal nerve.

## Case Presentation

**Figure 1:**
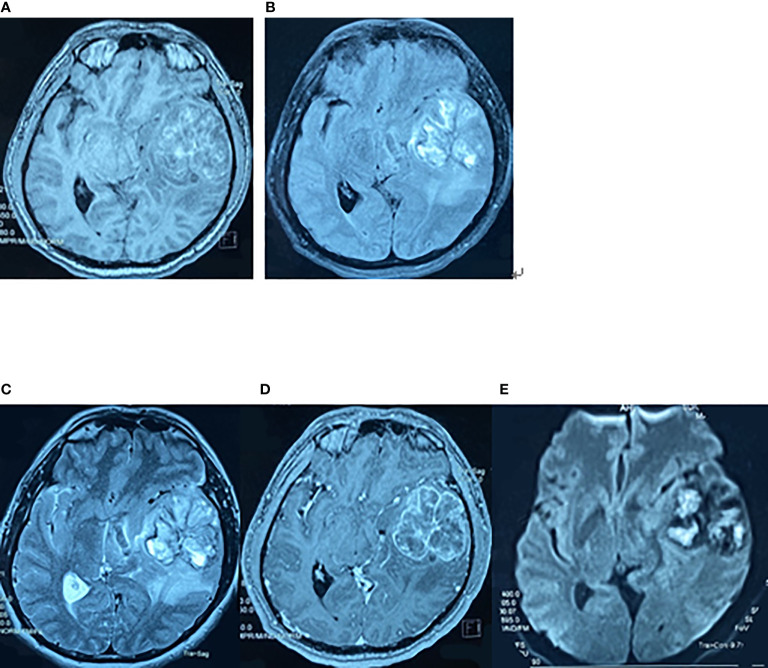
Preoperative brain MRI findings on May 7, 2017. The axial planes of preoperative MRI of the brain showing a 6-cm heterogeneous mass in the left superior temporal lobe. **(A)** T1-weighted MRI. **(B)** T2-weighted fluid-attenuated inversion recovery (FLAIR) MRI. **(C)** T2-weighted MRI. **(D)** Enhanced T1-weighted MRI. **(E)** Diffusion-weighted MRI.

**Figure 2:**
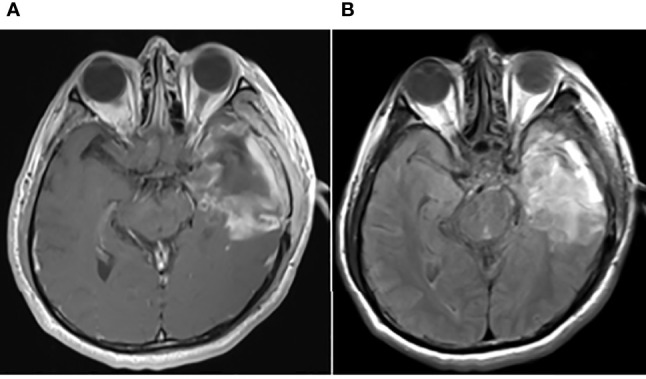
Postoperative brain MRI findings on May 31, 2017. The axial planes of postoperative brain MRI showed that the tumor had been removed. **(A)** Enhanced T1-weighted MRI. **(B)** T2-weighted FLAIR MRI.

On first admission (May 2017), a 47-year-old man presented with a 3-month history of headache, nausea, vomiting, and memory loss. On May 7, 2017, craniocerebral magnetic resonance imaging (MRI) revealed space-occupying lesions in the left temporal lobe (**Figure 1**). On May 9, 2017, the patient underwent craniotomy for left temporal lobe tumor resection. Intraoperatively, the tumor was found to have a clear capsule and a distinct boundary with the surrounding brain tissue. MRI was performed during the operation to confirm total tumor resection. The pathological diagnosis was GBM, and the patient’s symptoms were relieved. Immunohistochemistry (IHC) demonstrated the following: oligodendrocyte lineage transcription factor-2 (Olig-2) positive, α-thalassemia/mental-retardation-syndrome-X-linked gene (ATRX) positive, glial fibrillary acidic protein (GFAP) positive, isocitrate dehydrogenase 1 (IDH-1) wild-type, O6-methylguanine-DNA-methyltransferase (MGMT) protein 80% positive, P53 10% positive, programmed cell death 1 (PD-1) scattered cells positive, programmed cell death-ligand 1 (PD-L1) partial positive, and Ki-67 30% positive in tumor cell nuclei, indicating high mitotic activity. On May 31, 2017, postoperative reexamination of brain MRI showed that the tumor had been removed (**Figure 2**). Intensity-modulated radiation therapy (IMRT) (6,000 cGy total in 28 fractions) to the tumor bed was performed from June 5, 2017, to July 13, 2017. The patient was treated with temozolomide (TMZ) chemotherapy (75 mg/m^2^/day) as an adjunct to radiotherapy and then sequentially (first cycle 150 mg/m^2^/day, then 200 mg/m^2^/day for 5 days, per cycle of 28 days for 6 cycles) between June 5, 2017, and December 9, 2017. There were no obvious signs of recurrence or metastasis on craniocerebral MRI every 3 months after the operation.

**Figure 3:**
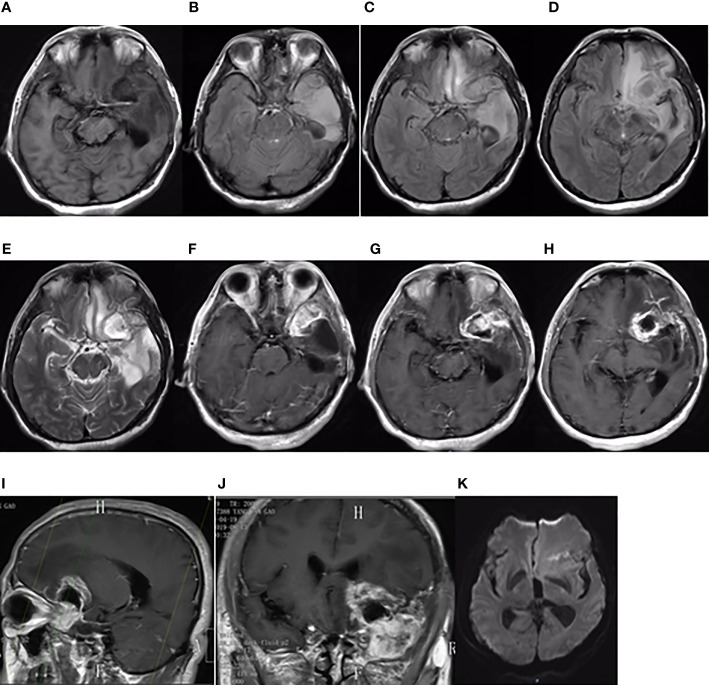
Brain MRI findings on August 13, 2019. Brain MRI showing a new focus in the postoperative residual cavity of the left temporal lobe. **(A)** The axial plane of T1-weighted MRI. **(B–D)** The axial planes of T2-weighted FLAIR MRI. **(E)** The axial plane of T2-weighted MRI. **(F–H)** The axial planes of enhanced T1-weighted MRI. **(I, J)** The sagittal and coronal planes of enhanced T1-weighted MRI. **(K)** The axial plane of diffusion-weighted MRI.

On second admission (August 13, 2019), MRI demonstrated recurrence of the tumor in the postoperative residual cavity (**Figure 3**). The tumor in the left frontotemporal lobe was removed on August 21, 2019. Intraoperatively, the tumor was located in the left temporal pole with an unclear boundary and no clear boundary with brain tissues. Postoperative pathology revealed GBM, World Health Organization (WHO) grade IV. IHC demonstrated the following: Olig-2, ATRX and GFAP positive, IDH-1 wild-type, MGMT protein revealed 10% positive, P53 weakly positive in partial cells, and Ki-67 revealed 20% positive. Postoperative genetic tests revealed the following: MGMT promoter was not methylated; 1p/19q genes intact; no mutations in IDH-1 R132, IDH-2 R172, or telomerase reverse transcriptase (TERT) C250T or BRAF V600E; and TERT C228T mutation. Antiepileptic drugs were not given after the two operations, and radiotherapy and chemotherapy were not performed after recurrence.

**Figure 4:**
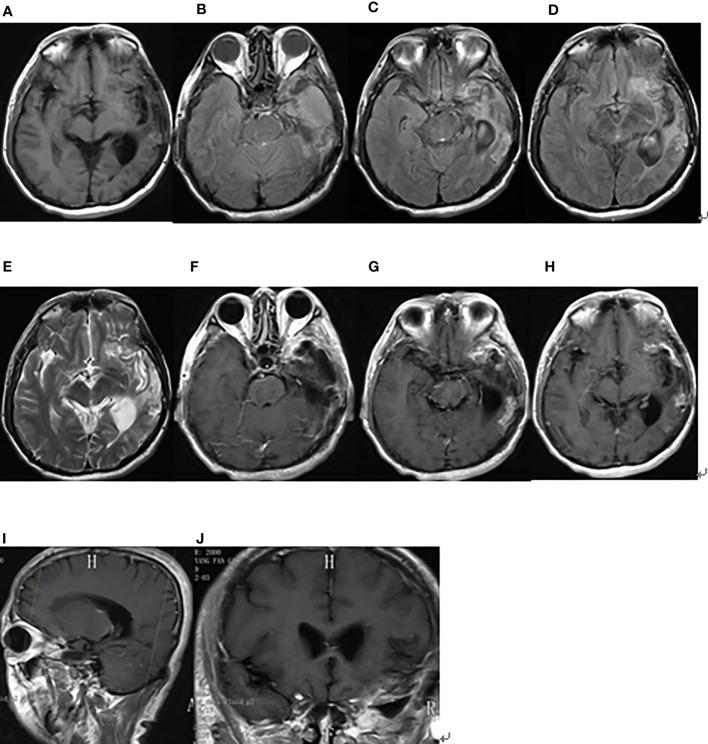
Brain MRI findings after the second surgery on December 4, 2019. **(A)** The axial plane of T1-weighted MRI. **(B–D)** The axial planes of T2-weighted FLAIR MRI. **(E)** The axial plane of T2-weighted MRI. **(F–H)** The axial planes of enhanced T1-weighted MRI. **(I, J)** The sagittal and coronal planes of enhanced T1-weighted MRI.

**Figure 5:**
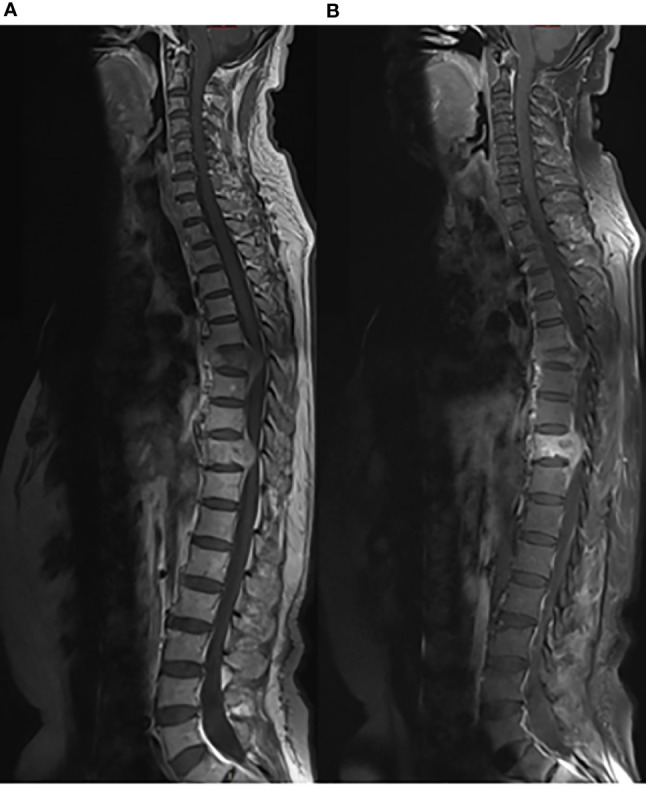
Whole spinal MRI findings on December 12, 2019. Sagittal planes of T1-weighted MRI of the whole spine showed metastases of the T7 and T10 vertebrae. **(A)** T1-weighted MRI. **(B)** Enhanced T1-weighted MRI.

**Figure 6:**
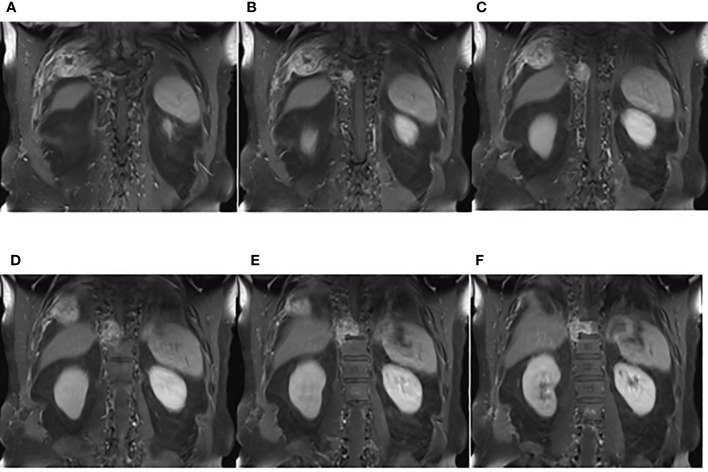
The coronal plane of MRI findings on December 6, 2019. **(A**–**F)** Metastasis of the thoracic vertebrae and chest wall on the coronal planes of enhanced T1-weighted MRI.

**Figure 7:**
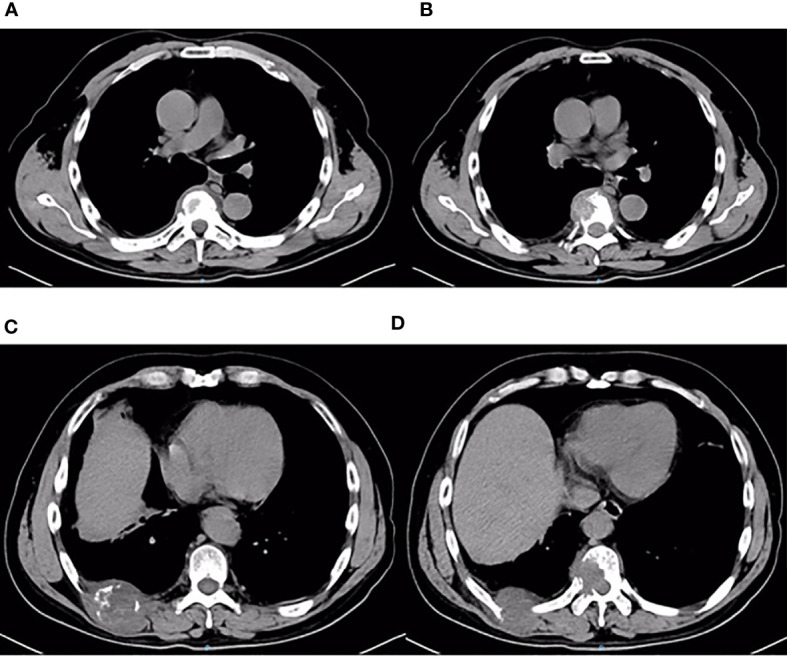
Thoracic and abdominal CT findings on December 6, 2019. Thoracic and abdominal CT revealed multiple metastases of the chest wall and thoracic vertebrae **(A**–**D)**. Metastases of the third anterior ribs on the left, T7 vertebrae and its appendages, ninth posterior ribs on the right, and T10 vertebrae and its appendages.

**Figure 8:**
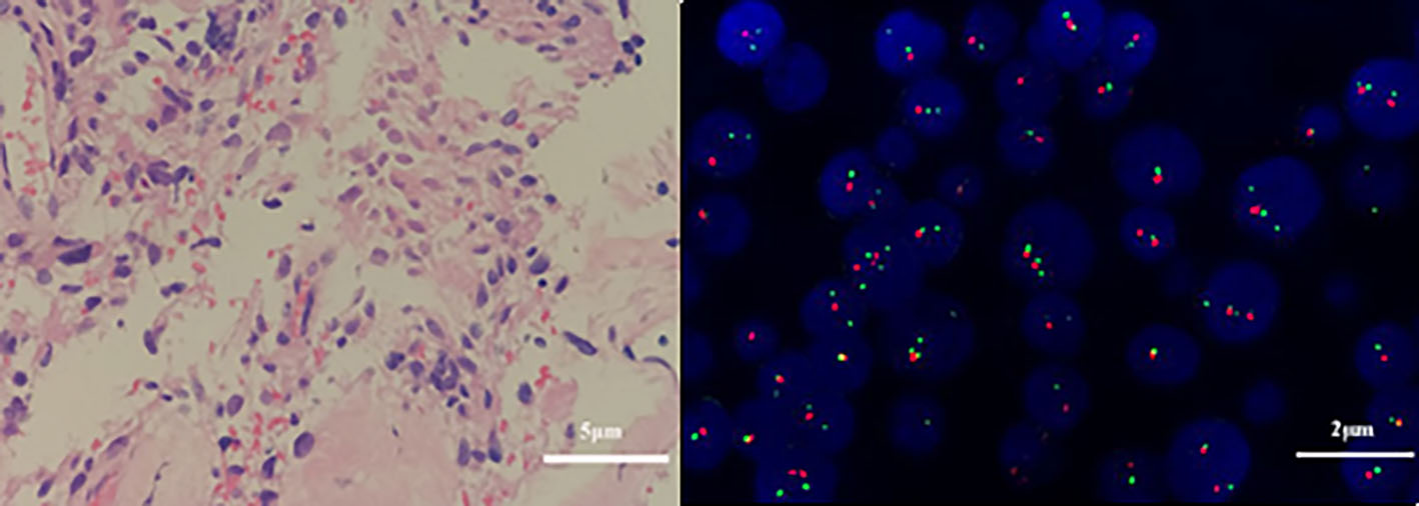
Pathological and genetic tests results of the fibrous tissue obtained on December 10, 2019. Atypical cells in the fibrous tissue, necrosis, and obvious cell atypia, in accordance with GBM, WHO grade IV. **(A)** Glial fibrillary acidic protein (GFAP) positivity. (hematoxylin and eosin, original magnification × 400). **(B)** Genetic tests revealed that the 1p/19q genes were intact (× 1000).

On third admission (November 2019), the patient presented with a 1-month history of back pain affecting sleep and aggravating in the recumbent position, with a numerical rating scale (NRS) score for the first 24 h of 4. On December 4, 2019, he underwent brain MRI. The intracranial conditions are shown in **Figure 4**. To identify postoperative changes and residual enhancing disease, an enhanced MR scan proved to be extremely valuable for assessing gross residual tumor when performed on postoperative 72 h; this timing avoided surgically induced contrast enhancement (8–10). The diffusion-weighted imaging (DWI) can be helpful in determining whether new enhancement developing in the subsequent weeks or months is postoperative changes or tumor recurrence (11, 12). Since craniocerebral MRI was not performed 72 h after the second surgery, it is difficult to identify the postoperative changes, residual tumors, or recurrence. MRI of the whole spine showed metastases of the T7 and T10 vertebrae and right posterior chest wall (**Figures 5**, **6**), while thoracic and abdominal computed tomography (CT) on December 6, 2019, showed the metastases of the ninth posterior ribs on the right, third anterior ribs on the left, and T7 and T10 vertebrae and their appendages (**Figure 7**). To determine the nature of the ninth posterior costal mass, CT-guided fine needle biopsy of the ninth posterior costal mass was performed on December 6, 2019. Postoperative pathology and IHC revealed the following: GBM (**Figure 8**), WHO grade IV, Olig-2, ATRX and GFAP positive, IDH-1 wild-type, P53 revealed 10% positive, Ki-67 revealed 10% positive. Vimentin and S-100 positive, CD31 and CD34 vascular positive, CK widely and Smooth Muscle Actin (SMA) negative. Genetic tests revealed that the MGMT promoter was not methylated, and 1p/19q genes were intact. The IHC and genetic test results are summarized in **Table 1**. On December 20, abscission cytological examination of the lumbar puncture was negative. To relieve pain and control the disease, IMRT (6145 cGy total in 28 fractions) was performed on the target areas (right ninth posterior costal soft tissue metastases; T7, T8, and T9 prevertebral soft tissue lesions; and T10 right vertebral soft tissue metastases), and the patient received TMZ synchronous chemotherapy (75 mg/m^2^/day). Oral analgesics were recommended during treatment from December 2019 to January 2020. At the end of treatment, the patient’s symptoms were slightly relieved, but there was still pain, with an NRS score of 2. The patient did not come for further consultation for personal reasons. According to a telephone follow-up, the patient’s pain worsened again in August 2020, and he received analgesic treatment at a local hospital. He eventually developed cachexia and died of systemic failure in December 2020. The progression-free survival (PFS) period was 27 months, and the overall survival (OS) period was 43 months.

**Table 1:** Summary results of immunohistochemistry and genetic tests.

BIOMARKER	2017.05.16 First surgical specimen	2019.08.28 Second surgical specimen	2019.12.10 Needle biopsy specimen
**IHC**			
Olig-2	positive	positive	positive
ATRX	positive	positive	positive
GFAP	positive	positive	positive
IDH-1	wild-type	wild-type	wild-type
MGMT	80% positive	10% positive	–
P53	10% positive	weakly positive	10% positive
PD-1	scattered cells positive	–	–
PD-L1	partial positive	–	–
Ki-67	30% positive	20% positive	10% positive
Vimentin	–	–	positive
S-100	–	–	positive
CD31	–	–	vascular positive
CD34	–	–	vascular positive
CK Widely	–	–	negative
SMA	–	–	negative
**GENETIC TEST**			
MGMT methylation	–	negative	negative
1p/19q	–	intact	intact
IDH-1 R132	–	not mutated	–
IDH-2 R172	–	not mutated	–
TERT C250T	–	not mutated	–
TERT C228T	–	mutated	–
BRAF V600E	–	not mutated	–

IHC, immunohistochemistry; Olig-2, Oligodendrocyte Lineage Transcription Factor-2; ATRX, α-thalassemia/mental-retardation-syndrome-X-linked gene; GFAP, Glial Fibrillary Acidic Protein; IDH-1, Isocitrate-dehydrogenase1; MGMT, O6-methylguanine-DNA-methyltransferase; PD-1, Programmed Cell Death1; PD-L1, Programmed cell death-Ligand 1; SMA, Smooth Muscle Actin; TERT, Telomerase reverse transcriptase; “-”, this test was not performed.

## Discussion

GBM is a highly aggressive tumor, comprising 56% of gliomas, which account for 14.7% of all primary central nervous system (CNS) tumors and 47.7% of all primary malignant CNS tumors (2). Surgery is the first-line treatment for GBM, and gross total resection improves survival (13). Currently, GBM is considered one of the most difficult tumors to treat *via* neurosurgical management (14). Postoperative radiotherapy plus concomitant and adjuvant chemotherapy with TMZ has also been the standard treatment (15). GBM has a very poor outcome, as the estimated median survival period is 12–15 months (2, 16), and the 5-year survival rate is 5.6% (17).

Although GBM cells can pass through the BBB and be disseminated throughout the peripheral blood (7), extracranial metastasis of GBM is rare, with a reported incidence of approximately 0.4% to 2% (6, 18, 19). This rare phenomenon is also attributed to the absence of lymphatics in the brain (20). Extraneural metastasis of primary CNS neoplasms typically occurs after a median interval of 2 years from diagnosis (21); patients with GBM do not survive long enough to develop such metastases (16, 22), in which not enough time is available for neoplastic cells to metastasize to extracranial organs (19, 23). The most common metastatic sites include the lungs, pleura, lymph nodes, soft tissues, glands, and bones (19, 21, 24, 25). There may be several mechanisms through which GBM escapes the CNS; hence, the locations of the metastases widely vary and include the following: vascular invasion, cranial nerve perineural spread, lymphatic spread, direct invasion, or iatrogenic spread into soft tissue (19). Extracranial GBMs are most commonly found in patients who previously underwent invasive surgery or biopsy or received ventriculoperitoneal shunts, which create iatrogenic access to extracranial structures and disrupt the BBB (19); one clear example of this possible pathway is the migration of GBM cells *via* the movement of CSF through shunts to the peritoneum or pleura (26). Previous studies have highlighted that, presumably, a tumor gains access to lymphatics by dural or scalp extension through a surgical defect (27, 28). Additionally, the spontaneous trafficking of GBM cells out of the brain may occur *via* the CSF, lymph drainage that ends in the lymph nodes or blood, and invasive procedures increase the access of tumor cells to metastasize (23). However, this does not explain lymph node metastasis without any invasive manipulation, in which case, intrinsic factors of the tumor may play a role (29). Later studies indicated a lymphatic pathway of GBM metastasis (30). Malte Mohme et al. described that extracranial metastasis of GBM may also be related to genetic drivers and immune escape (7). GBM metastasizing to the distal limbs has not been reported. This indicates that the main vascular route of GBM metastasis is reflux into the systemic venous system, as valves in the peripheral veins prevent such reflux, whereas arteries have no valves and reach the distal limbs (19).

Approximately 10.5% of extracranial metastases of GBM involve multiple metastases, with or without intracranial recurrence (31). The most common sites of bone metastases are the vertebral spine and the thoracic cage (6, 25). Bone metastases of malignant gliomas should show lytic or sclerotic features on neuroimaging, and they are usually reported as multiple lesion spread (21, 32), as observed on an image from the patient described herein. The GBM in our patient could have metastasized *via* multiple possible pathways, including those described above. However, interestingly, the two metastases that were seen on imaging in December 2019 and the soft tissue metastasis in the ninth posterior costal ribs and T10 metastasis (**Figure 6**) in our case were consistent with the shape of the ninth intercostal nerve; therefore, we made the bold assumption that GBM could migrate through the nerve root or intercostal nerve. Multiple metastases of the T7 and T10 vertebrae and their appendages may have been caused by tumor invasion into the dura mater. As current studies have shown that there are few cases of nerve root metastasis of malignant tumors (33), so the metastasis in our case is a coincidence or a fact. More clinical cases are needed to confirm this finding.

The survival time of GBM patients is related to many factors. Research has shown that GBM with extracranial metastases most often involves the temporal lobe, either coincidentally or factually (31). For IDH-1 wild-type GBM, which accounts for almost 90% of GBMs, the median survival period of patients receiving the Stupp protocol is 15 months (34). MGMT promoter methylation, P53 and EGFR negativity and IDH 1/2 mutations have shown to be associated with improved OS and PFS in patients with GBM; ATRX and VEGF positivity are associated with worse outcome (34–40). At present, there are still many unknown molecular pathological mechanisms; thus, treatment has not progressed much over recent decades (41). Recently, a multitude of novel therapies including PD-L1/PD-1 inhibitors to maximize survival benefits and move treatment towards precision medicine have achieved effective responses (42–45). We report this case of GBM with multiple metastases of the left temporal lobe, with which wild-type IDH-1, ATRX and P53 positivity, affected the outcomes.

## Conclusion

Although the number of reports of GBM metastases has increased over time, the pathogenesis of such metastases remains elusive, and this diagnosis remains rare (41, 46). Our case report indicates a new pathway of metastasis. The extracranial metastasis in this patient may have occurred through the nerve root or intercostal nerve, but more reports of GBM metastases are needed to confirm this finding.

## Data Availability Statement

The original contributions presented in the study are included in the article/supplementary material. Further inquiries can be directed to the corresponding authors.

## Ethics Statement

The studies involving human participants were reviewed and approved by the Ethics Committee of Weifang People’s Hospital. Written informed consent for participation was not required for this study in accordance with the national legislation and the institutional requirements.

## Author Contributions

WZ, YYC, and FRH performed the chemoradiotherapy in this case. WZ, XLW, and XXW drafted the manuscript and performed the literature review. YL and YJC retrieved the clinical and the image information, GYH and YXZ retrieved and analyzed the pathological information. FRH and YXZ conceived, designed, and supervised this study. The paper properly credits the meaningful contributions of the coauthors and coresearchers. All authors have been personally and actively involved in substantial work leading to the publication of paper and take public responsibility for its content. All authors contributed to the article and approved the submitted version.

## Conflict of Interest

The authors declare that the research was conducted in the absence of any commercial or financial relationships that could be construed as a potential conflict of interest.

## Publisher’s Note

All claims expressed in this article are solely those of the authors and do not necessarily represent those of their affiliated organizations, or those of the publisher, the editors and the reviewers. Any product that may be evaluated in this article, or claim that may be made by its manufacturer, is not guaranteed or endorsed by the publisher.
